# Multifractal Characteristics of Geomagnetic Field Fluctuations for the Northern and Southern Hemispheres at Swarm Altitude

**DOI:** 10.3390/e23050558

**Published:** 2021-04-30

**Authors:** Benjamín Toledo, Pablo Medina, Sylvain Blunier, José Rogan, Marina Stepanova, Juan Alejandro Valdivia

**Affiliations:** 1 Departamento de Física, Facultad de Ciencias, Universidad de Chile, Las Palmeras 3425, Ñuñoa, Santiago 8370415, Chile; btoledoc@uchile.cl (B.T.); jrogan@uchile.cl (J.R.); alejo@uchile.cl (J.A.V.); 2 Centro para el Desarrollo de la Nanociencia y la Nanotecnología (CEDENNA), Avenida Libertador Bernardo O’Higgins No. 3363, Estación Central, Santiago 8370415, Chile; 3 Departamento de Física, Facultad de Ciencias, Universidad de Santiago de Chile, Avenida Libertador Bernardo O’Higgins No. 3363, Estación Central, Santiago 8370415, Chile; marina.stepanova@usach.cl

**Keywords:** multifractal, magnetosphere, auroral electrojet index

## Abstract

This paper explores the spatial variations of the statistical scaling features of low to high latitude geomagnetic field fluctuations at Swarm altitude. The data for this study comes from the vector field magnetometer onboard Swarm A satellite, measured at low resolution (1 Hz) for one year (from 9 March 2016, to 9 March 2017). We estimated the structure-function scaling exponents using the *p*-leaders discrete wavelet multifractal technique, from which we obtained the singularity spectrum related to the magnetic fluctuations in the North-East-Center (NEC) coordinate system. From this estimation, we retain just the maximal fractal subset, associated with the *Hurst* exponent *H*. Here we present thresholding for two levels of the Auroral Electrojet index and almost the whole northern and southern hemispheres, the *Hurst* exponent, the structure-function scaling exponent of order 2, and the multifractal *p-exponent* width for the geomagnetic fluctuations. The latter quantifies the relevance of the multifractal property. Sometimes, we found negative values of *H*, suggesting a behavior similar to wave breaking or shocklet-like propagating front. Furthermore, we found some asymmetries in the magnetic field turbulence between the northern and southern hemispheres. These estimations suggest that different turbulent regimes of the geomagnetic field fluctuations exist along the Swarm path.

## 1. Introduction

Nowadays, understanding ionospheric activity is becoming of great economic importance because it has direct consequences for applications that rely on communication and positioning such as air traffic control, city traffic control, ship navigation, national security issues, citizen users, etc. The ionospheric activity seems to be affected by the plasma and electromagnetic field dynamics, which requires understanding their behavior at different scales.

In this respect, the Earth’s ionosphere is typically described as a complex and turbulent region where the plasma and electromagnetic field dynamics [1,2,3] display spatio-temporal multiscale phenomena [4,5,6,7,8] with intermittent self-similar fluctuations that suggest a nonlinear cascading process [9,10,11,12].

This region plays a significant role in the solar wind–magnetosphere–ionosphere coupling and reflects the complex and turbulent evolution present in the system. It is well known that the system is driven by the turbulent solar wind (e.g., see [13], and references therein), whereas the magnetosphere contains several regions where complex and turbulent phenomena prevail (e.g., see Refs. [14,15,16,17], and references therein). However, there seems to be a global organization of at least part of the system [18,19] as reflected in the relative success of system science approaches to find how these fluctuations couple through the different regions [20] and provide reliable forecasts. For example, ionospheric indexes and magnetic field patterns related to solar wind parameters have been identified  [21,22,23]. Although several suggestions have tried to put forward an explanation for these seemingly contradicting observations, the self-organization paradigm, in which a low dimensional global state can live together with self-similar fluctuations, that is usually multifractal, may be a viable possibility [24,25]. Hence, characterizing the multifractal nature of these plasma and electromagnetic fluctuations, and particularly their spatial variation becomes of relevance to advance our understanding of the system. The ionosphere as measured by the Swarm satellites provides a reliable place to do this.

In this region, magnetosphere plasma instabilities and the production, loss, and transport of particles can strongly affect the ionospheric processes. These may be responsible for part of the turbulence phenomena at these scales. The ionospheric convection depends on various parameters, for example, the strength, and orientation of the interplanetary magnetic field (IMF) [3,26]. When the IMF points northward, a four-cell convection pattern is observed across the polar cap. For southward IMF, a two-cell convection pattern with anti-sunward convection is detected. In the range between $10^{2}$ and $10^{3}$ km at high latitudes, using structure-function analysis, Pulkkinen et al. [27] showed evidence of the scale-free structure of the spatio-temporal scaling properties of the horizontal magnetic field fluctuations in the auroral region. Equivalent results were found in [28] considering the first-order structure-functions of the ionospheric plasma velocity. Similarly, several works suggested the intermittent turbulent nature of the small-scale variations of the ionospheric electric field [1,4,11,12]. In [8,29], these were confirmed through data analysis, demonstrating that the turbulent electric and magnetic field fluctuations were self-similar and intermittent in the ionosphere.

Despite the great amount of evidence about the self-similar and intermittent behavior of the electromagnetic field fluctuations, there is a need to characterize the multifractal nature of these fluctuations through its multifractal spectra; and in particular its spatial variation in the northern and southern hemispheres, for quiet and active periods.

Hence, here we will concentrate on the study of the complex behavior of the ionospheric magnetic field at the Swarm satellite height, as characterized by the multifractal spectra of the magnetic field fluctuations. Several multifractal techniques have been used in the past, such as the detrended multifractal formalism  [9,10], but here we will use the *p*-leaders wavelets technique because of its quantitative robustness. This technique will not only allow us to obtain multiple measures of the spectra, such as the Hurst exponent or the second-order structure-function, but also the complete spectra in a reliable manner, and in particular, the spectrum width that characterizes how multifractal can become the system, as opposed to monofractal [30,31].

In particular, we analyze the spatial variation of the multifractal features associated with the complex state that appears under quiet and active conditions, as determined by the Auroral Electrojet (AE) index level. It is common to characterize the geomagnetic activity in the polar region with this index. This index is derived from the difference between the upper and lower part of the envelope of the magnetic field measured in stations near the North Polar Circle at ground altitude [32]. There is no consistent definition of active and quiet periods for the geomagnetic activity, so many threshold values have been used in the past. Probably this is related to the self-similar (power-law distribution) event statistics of the AE Index [25], so defining such a threshold value may be difficult to do. For example, De Michelis et al. [9] separated quiet periods with AE<60 nT and active periods with AE>80 nT. Another consideration has been taken to define high-intensity, long-duration, continuous AE activity (HILDCAA) events that have been proposed by [33]. During these events, AE is maintained above 200 nT. This definition has been used by [34] and later by [35]. Therefore, here we use the arbitrary value of AE=200 nT to separate the quiet and active periods, but of course, other values can be used. Below, we will provide an additional argument for the AE=200 nT threshold.

Following De Michelis et al. [10], to characterize the geomagnetic field fluctuations, we use the data provided by the Swarm mission [36], which consists of three identical satellites (A, B, C) flying in a near-polar, circular orbit. With the data provided by the satellites, it is possible to study the spatial variation, filtered by quiet and active times, of the multifractal spectrum of the magnetic field fluctuations. We will see that the main properties of the spectra vary between active and quiet times, and for southern and northern hemispheres. These calculations should help to provide some modeling restrictions about the multifractal behavior present in the ionospheric magnetic field fluctuations, that seems to occur naturally in complex systems, and suggest the existence of an out-of-equilibrium dynamically global state with an underlying multifractal behavior.

This paper is written as follows. In Section 2, we present the multifractal formalism based on the discrete wavelet transform. In Section 3, we describe the data processing we used in our approach, and we present the main findings of our work. Finally, in Section 4, we present a discussion and final remarks.

## 2. Multifractal Formalism

It was observed that some measurements made in the context of fully-developed turbulence deviate from the Kolmogorov theory (K41) on homogeneous and isotropic turbulence [37]. To improve the processing of this data, multifractal formalisms (MF) were developed. One of the first successful multifractal approaches to singular probability measures [38] was based on the continuous wavelet transform [39]. From this success, multifractal wavelet methods have been applied not only to fully developed turbulence, but also to econophysics, meteorology, physiology, and DNA sequences [40] to cite a few applications.

Now we will describe the MF used in our data analysis [41]. Let us take a time-series x(t), and let us define its local regularity around $t_{0}$ by its Hölder exponent [42] $h(t_{0})⩾0$, which is given as the largest *h* such that,

(1) $$\begin{array}{c} |x\left(t\right)-P\left(t-t_{0}\right)|\leq C|t-t_{0}{|}^{h}, \end{array}$$

where $P(t-t_{0})$ is a polynomial of degree *n*, such that n<h<n+1 and C>0 [43] (let us note that this is related to the general results of Weierstrass in approximation theory). Now, let us denote by $E_{h_{0}}$ the time-series support, with an associated Hölder regularity $h_{0}$, such that $1\geq \dim\left(E_{h_{0}}\right)\equiv D\left(h_{0}\right)$ is the Hausdorff dimension of $E_{h_{0}}$. Since the measurement process resulting in a time-series x(t) is not mathematically exact, it is not possible to measure D(h) directly, therefore here we use the MF procedure. We will use the *p*-leaders discrete wavelet multifractal approach [44] to set up the MF, which has well established properties to analyze the multifractal spectrum of discrete time-series. As usual in this approach we will take the function ψ as the mother wavelet and define

(2) $$\begin{array}{c} \psi_{j,k}\left(t\right)=2^{-j/2}\psi \left(2^{-j}t-k\right),\;j,k\in Z, \end{array}$$

as the dilated and translated analyzing wavelets, which form an orthonormal basis of $L^{2}\left(R\right)$, i.e., a normed vector space with Euclidean norm. Here, the integer *k* denotes the discretized time translation, and *j* the time scale. An important feature of the mother wavelet is its number of vanishing moments, which is defined as $N∋N_{\psi }\geq 1$ such that $\forall s=0,\ldots ,N_{\psi }-1$,

(3) $$\begin{array}{c} \int_{R}t^{s}\psi \left(t\right)\;dt=0 \end{array}$$

and

(4) $$\begin{array}{c} \int_{R}t^{N_{\psi }}\psi \left(t\right)\;dt\neq 0. \end{array}$$

**Figure 1:**
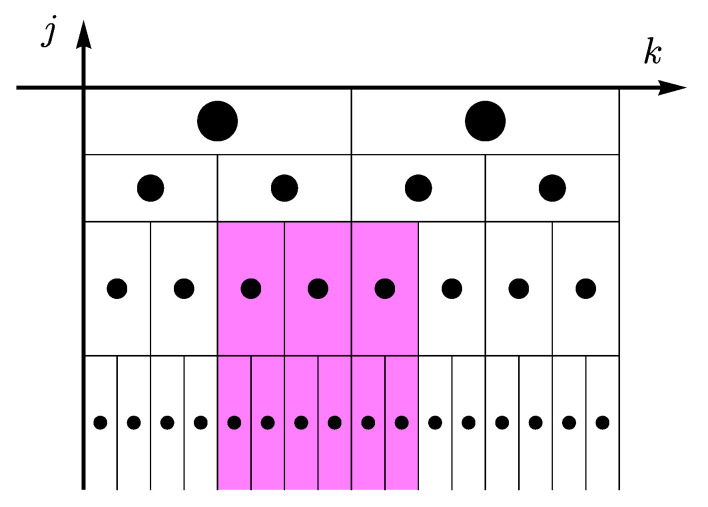
Space-scale plane and the dyadic tree. Discrete wavelet coefficients d(j,k) are represented by dots (•), and the dyadic interval $\lambda_{j,k}$ by the surrounding rectangle. The shaded area sketches the subset $3\lambda_{j,k}$ associated with a wavelet *p*-leader $L_{x}^{\left(p\right)}\left(j,k\right)$.

This property is very useful in our analysis since it provides an automatic detrending for signals well approximated by polynomials at scales much greater than fluctuation scales. It is similar to a high-pass filter, but consistent with Equation (1). Let

(5) $$\begin{array}{c} d_{x}\left(j,k\right)=\int_{R}x\left(t\right)\psi_{j,k}\left(t\right)\;dt, \end{array}$$

denote the ($L^{1}$-normalized, i.e., we use the taxicab norm, which is maximal) discrete wavelet transform coefficients of x(t), where *j* is related to the time scale $\tau =2^{j}$, and *k* to the time translation. The wavelet *p*-leaders $L_{x}^{\left(p\right)}\left(j,k\right)$ are multi-resolution quantities defined as

(6) $$\begin{array}{c} L_{x}^{\left(p\right)}\left(j,k\right)={\left(\sum_{\lambda^{\prime }\subset 3\lambda_{j,k},\;j^{\prime }\leq j}{\left|d_{x}\left(\lambda^{\prime }\right)\right|}^{p}\right)}^{1/p}, \end{array}$$

where $\lambda_{j,k}=\left[2^{j}k,2^{j}\left(k+1\right)\right)$ and $3\lambda_{j,k}={⋃}_{m\in \left\{-1,0,1\right\}}\lambda_{j,k+m}$. Here, we chose p=1, that is related to the $L^{1}$-norm, because it is robust against outliers. Sometimes, researchers denote them as “1-leaders MF”, to distinguish different values of *p*. Hence, for a given *k* and *j*, the summation is taken over the region shown in the schematic representation of Figure 1.

For a fixed time scale $\tau =2^{j}$, the time averages of the $q^{\mathrm{th}}$ powers of the $L_{x}\left(j,k\right)\equiv L_{x}^{\left(1\right)}\left(j,k\right)$ are referred to as the structure-functions

(7) $$\begin{array}{c} S^{L}\left(j,q\right)=\frac{1}{n_{j}}\sum_{k=1}^{n_{j}}L_{x}^{q}\left(j,k\right), \end{array}$$

where $n_{j}$ is the number of *p*-leaders $L_{x}\left(j,k\right)$ available at scale $2^{j}$. We define ξ(q) as

(8) $$\begin{array}{c} S^{L}\left(j,q\right)=F_{q}\;2^{j\xi (q)}, \end{array}$$

where $F_{q}$ is some constant. It can be shown that

$$\begin{array}{c} S^{L}\left(j,q\right)\;\to \;\lim_{\Delta t\to 0}\int {\left|x\left(t+\Delta t\right)-x\left(t\right)\right|}^{q}dt, \end{array}$$

in the limit where $2^{j}\to 0$ [44] (j<0). We should compare this expression with

$$\begin{array}{c} \lim_{\Delta x\to 0}\int_{R}{\left|v\left(x+\Delta x,t\right)-v\left(x,t\right)\right|}^{q}\;dt, \end{array}$$

that relates the MF to structure-functions based on velocity increments, which are an essential ingredient of the Parisi and Frisch conjecture for turbulence [37,45]. Hence, the MF provides a robust generalization of such structure-functions. The MF procedure is then completed by introducing the Legendre transformation that establishes a link between ξ(q) and D(h) as

(9) $$\begin{array}{c} D\left(h\right)=1+\min_{q}\left(qh-\xi \left(q\right)\right). \end{array}$$

Therefore, an MF consists of obtaining an approximation of D(h) from estimations of ξ(q) [46].

A last issue that should be remembered is the minimal Hölder regularity to apply the MF, which imposes the condition [47]

(10) $$\begin{array}{c} h_{m}=\underset{2^{j}\to 0}{lim inf}\left(\frac{\ln(\sup_{k}\left|d_{x}\left(j,k\right)|\right)}{\ln2^{j}}\right)>0, \end{array}$$

so that it requires a bounded time-series [43]. If this is not satisfied, a fractional integration has to be done [48]. The fractional integration of order η>0: $I^{\eta }$, of a function or measure *X*, is defined in the Fourier domain as (see [47] and references therein)

(11) $$\begin{array}{c} \hat{\left(I^{\eta }X\right)}\left(\zeta \right)={\left(1+|\zeta \right|}^{2}{)}^{\eta /2}\hat{X}\left(\zeta \right), \end{array}$$

where the hat ($\hat{\ldots }$) denotes Fourier transform. When η∈N, it reduces to the usual integral. The detailed derivation of the expressions recalled here is beyond the scope of this study, and the reader is referred to the books by Mallat [49] and Jaffard [50], and the citations in this section. For the numerical estimation of the multifractal quantities we used the MATLAB toolbox provided by Wendt [30,44,47,51], which uses Daubechies wavelets [52].

## 3. Data and Processing

**Figure 2:**
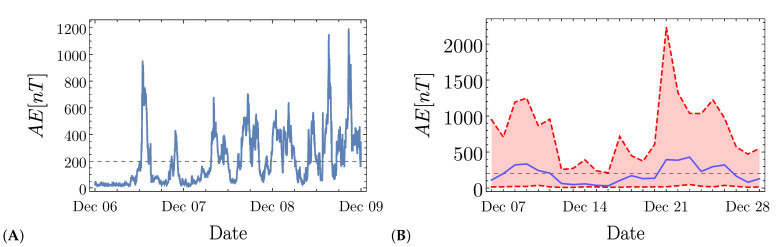
AE index time-series during period of Dec 6–Dec 29, 2016. In (**A**), we show the behavior of the AE index with a resolution of one minute for the three first days (Dec 6–Dec 8, 2016). In (**B**), we summarize the mean (blue solid line), the maximum, and the minimum of the variation of the AE index during a day (red area bounded by the maximum and the minimum values depicted by the red upper and lower lines). The black dashed horizontal line in both panels indicates the threshold AE=200 nT used in our approach.

The Auroral Electrojet index AE is obtained with minute resolution from the OMNI database provided by the National Aeronautics and Spatial Agency (NASA). Figure 2 shows an example of the AE index behavior. In panel (A), we show the AE index for three consecutive days (6 December–8 December 2016). It is possible to observe the intermittent dynamics of the AE index, displaying large fluctuations. As mentioned above, the event statistics of AE seem to follow a power-law distribution [25], so that a clear threshold value between quiet and active periods might be difficult to define. The dashed horizontal line indicates AE=200 nT, the value we used as the threshold that separates quiet and active times. In panel (B), we display the highest, lowest, and mean values of the AE index averaged to a 24 hour period, plotted from 6 December to 29 December 2016. Even at this scale, we observe large fluctuations of the values of the AE series. We note that the threshold of 200 nT is consistent with the daily averaged value of AE, so that in our study, we rate the magnetosphere as “active” when AE>200 nT and “quiet” otherwise.

To characterize the spatial variation of the intermittent magnetic field fluctuations, we use the magnetic field data from the Swarm mission [36], labeled as MAGx_LR_1B version 0501, which consists of CDF files with several time-series. A detailed description of each one can be found on the mission website [36]. We use data from the vector field magnetometer on the Swarm A satellite, measured at low resolution (1 Hz). For our analysis, we use the following parameters: Timestamp, Latitude, Longitude, B_NEC, and Radius. First, we calculated the Altitude Adjusted Corrected Geogmagnetic Coordinates [53], and proceed to split the data in chunks of 256 samples of consecutive points (a discussion on the time-series length can be found in [54]), which corresponds to about 4.27 min. The procedure described above performs better with time-series whose length is a power of 2. The multifractal analysis was done using the MATLAB toolbox provided by Wendt [51].

First, we need to find a common scaling region where $\log_{2}S^{L}\left(j,q\right)$ changes linearly with *j*, so that ξ(q) can be found. Furthermore, ξ(q) must satisfy the restrictions of being a monotonically increasing function of *q* and that the spectrum of singularities looks like an inverted parabola (concave), at least for a range of values of *q*. If these restrictions are not satisfied, probably the range of *j* was not chosen correctly. We use $N_{\psi }$ to maximize the number of instances that satisfy these restrictions. Here, $N_{\psi }$, in essence, provides an automatic detrending for signals well approximated by polynomials at scales much greater than the fluctuations we are interested in. In our case, this turns out to be $N_{\psi }=3$. Intervals that do not have at least a range of values of *q* that satisfies these restrictions are not considered.

**Figure 3:**
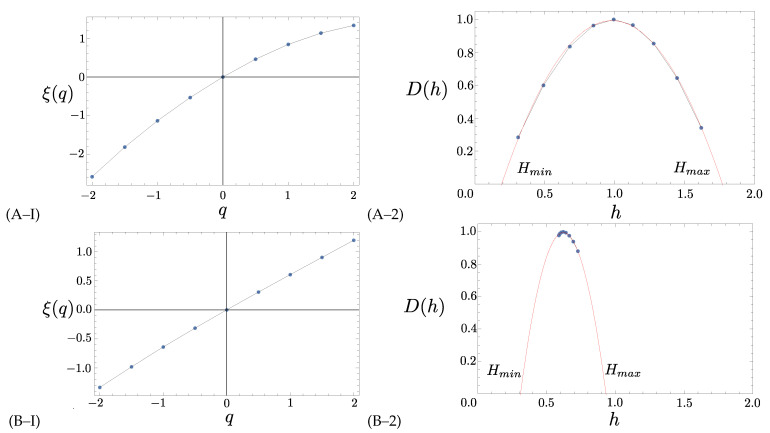
Multifractal 1-leaders evaluation for the center (C) component of the magnetic field in the NEC coordinate system at 2 instances during the satellite trajectory in the years 2016 and 2017. For the first instance (Swarm A satellite on 09/03/2016 at 03:00:48 with coordinate (36.905492,11.517977)), we show (**A–I**) ξ(q) and (**A–2**) the derived singularity spectrum D(h). Similarly, for the second instance (Swarm A satellite on 09/03/2017 at 10:48:13 with coordinate (46.188009,14.305622)), we show (**B–I**) ξ(q) and (**B–2**) the derived D(h). We denote *H* as the value of *h* at the maximum of D(h); and $\Delta H=h_{max}-h_{min}$, as the width of the extrapolated spectrum.

In Figure 3, we show how the analysis is applied to the center (C) component of the magnetic field in the NEC coordinate system, for two instances along the satellite trajectory. Here, we ask the code to find ξ(q), for −2≤q≤2, but only keep the values of *q* that satisfy the restrictions. The first instance (panel (A–I) and (A–2)) was chosen on March 9, 2016 at 03:00:48 when the satellite was above coordinate (lat,long) (36.905492,11.517977), while the second instance was picked on 9 March 2017 at 10:48:13 with coordinates (46.188009,14.305622). The length of the intervals used for the calculation is the same as discussed above, namely 256 consecutive values. First, we calculated ξ(q) for the two intervals (left panels of Figure 3). In these figures, the variation to the straight line represents a quantitative notion of multifractality, so that the bottom one (Figure 3B–I) is quite monofractal, while the top one (Figure 3A–I) is significantly more multifractal. To quantify the variation, we perform the Legendre transformation and compute the singularity spectrum (left panels) for the two instances and see that indeed the top one (Figure 3A–2) is much more multifractal than the bottom one (Figure 3B–2). From these spectra, we can define the Hurst exponent $H∼max_{D(h)}\left(h\right)$, and the singularity spectrum width $\Delta H=H_{max}-H_{min}$. These two values are provided by extrapolation (see references described above for details). The values *H* and ΔH will quantify the different intermittent regimes. For example, the larger the ΔH, the more multifractal the fluctuations are. Similarly, *H* quantifies the relevance of persistence vs. randomness of the main fractal component of the fluctuations. For H<0.5, the dynamic is virtually Brownian, with a slight but sensible tendency to be anti-persistent, which is reminiscent of a system trying to acquire statistical stability under a complex forcing environment. Note that in this case, the observed values of *H* are greater than zero ($H_{min}\geq 0$).

Another interesting measure is the value of ξ(2) that is directly related to the power-law index of the spectrum of the fluctuations. In this sense, the scaling exponent above or below 1 implies changes in self-correlation. A scaling exponent closer to zero indicates over-random coordination and a scaling exponent closer to 2 may indicate an over-regular coordination [55]. These three measures will be used to characterize the spatial variation of the intermittency of the magnetic field fluctuations in the northern and southern hemispheres using the Swarm satellite magnetic field data [36].

**Figure 4:**
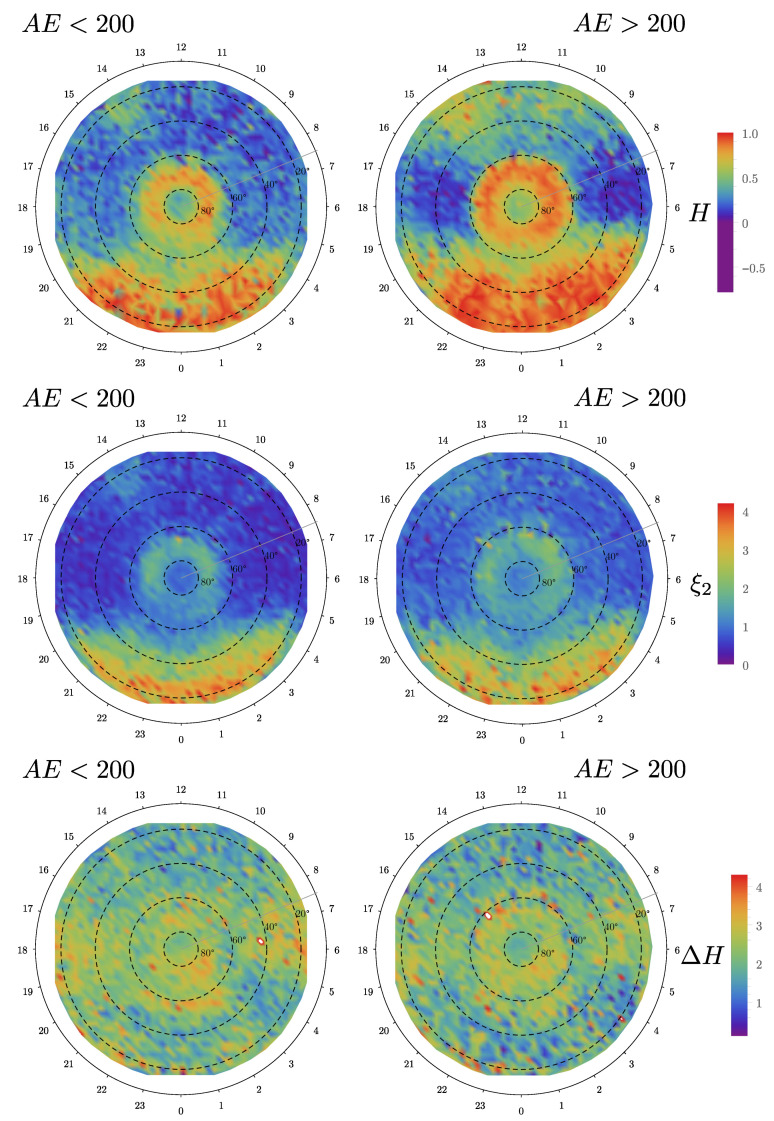
(**Top panels**) Hurst exponent (*H*), (**middle panels**) structure-function scaling exponent for q=2 ($\xi_{2}$), and (**bottom panels**) singularity spectrum width (ΔH) for the northern hemisphere, calculated for the center (*C*) coordinate. The left panels correspond to quiet conditions for threshold value AE<200, while the right panels correspond to active conditions for threshold value AE>200.

**Figure 5:**
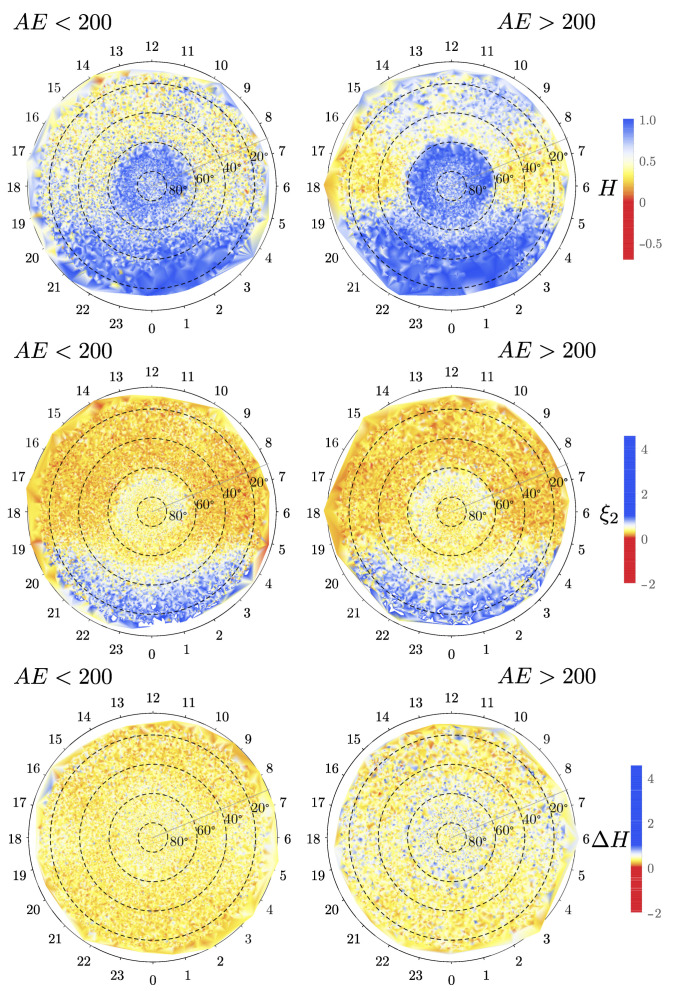
(**Top panels**) Hurst exponent (*H*), (**middle panels**) structure-function scaling exponent for q=2 ($\xi_{2}$), and (**bottom panels**) singularity spectrum width (ΔH) for the southern hemisphere, calculated for the center (*C*) coordinate. The left panels correspond to quiet conditions for threshold value AE<200, while the right panels correspond to active conditions for threshold value AE>200.

After finding a common scaling region, we obtained a total of 358,115 singularity spectra for the NEC magnetic field components spatially distributed over all latitudes and longitudes, and proceed to filtered by concavity (see Figure 3). Finally, when we already obtained the singularity spectra and related descriptors, we filtered by the two levels of AE index defined by the threshold value of 200 nT. This was done using the timestamp to match the data. The results that are shown below represent the spatial variation of these three measures for the center (C) component of the magnetic field fluctuations in the NEC coordinate system, after all the validation and filtering. We kept just 15,000 scattered points which were linearly interpolated to produce the usual density maps for the northern and southern hemispheres (see the six panels of Figure 4 and Figure 5). The other components, which are not shown here, display similar patterns.

We note from Figure 4, for quiet conditions AE<200 nT, that *H* is significant in the night side region for the whole latitude range, but also in the auroral region, above 60deg magnetic latitude, for all local times. This is an indication that the auroral region is remarkably different from more southern regions concerning dynamics and turbulent processes. These results can be contrasted by those obtained in Ref. [56] for the density and in Ref. [10] for the magnetic field fluctuations. In particular, the spatial patterns for the density, at different solar wind clock angles, do not have a clear north–south or dawn–dusk symmetry. In our case, the spatial pattern of *H* does appear to have a dawn–dusk symmetry. Additionally, in the dawn and dusk sectors, below 80deg magnetic latitude, we observe H<0.5, which implies that the dynamics is virtually Brownian, with a slight but sensible tendency to be anti-persistent, independently of activity. This is reminiscent of a system trying to acquire statistical stability under a complex forcing environment. It is interesting to note that *H* reaches negative values in several regions, particularly in the dawn and dusk sectors (below 80deg magnetic latitude) for active periods, which suggests a behavior similar to wave breaking or shocklet-like [57] propagating fronts. The fact that *H* becomes more negative in dusk and dawn during the active periods might have consequences for the predictability of the magnetic fluctuations in these regions. Note that there is a one-to-one correspondence between the structure-function scaling exponents of q=2 and the spectral scaling exponents. For quiet conditions, we see that we have steeper power-law power spectra at midnight, as opposed to dusk, dawn, and noon. For this measure, we also observe a remarkably different behavior in the auroral zone compared to the other regions. Similarly, the strength of the intermittent behavior, as characterized by ΔH varies significantly in magnetic latitude and magnetic local time, with a tendency for larger values (more intermittent) for dawn and dusk (ΔH∼3) and a smaller value for midnight and noon (ΔH∼1), at least below 80deg magnetic latitude. For active conditions, namely AE>200 nT, negative values of *H* become common for dawn and dusk, while its value seems to increase for noon. Under the same conditions, $\xi_{2}$ does not change significantly compared with the quiet conditions. Similarly, ΔH also varies significantly over magnetic latitude and magnetic local time as in the quiet case.

The same analysis is repeated for the southern hemisphere in Figure 5. Although we note that there are fewer measurements, we also note that *H* is consistently larger for active periods compared with quiet times, especially around midnight. This also occurs in the northern hemisphere. We note that the values of *H* in the southern hemisphere are relatively larger than for the northern hemisphere, especially for dawn and dusk, for both quiet and active times. The north–south asymmetry can also be observed in $\xi_{2}$, particularly for noon, dawn, and dusk. On the other hand, the strength of the multifractality, characterized by ΔH, seems to be larger in the northern hemisphere compared with the southern hemisphere. We conjecture that this asymmetry comes from the different amounts of particles and electromagnetic energy injected into these hemispheres, as suggested recently in Ref. [58], however, this needs further detailed studies.

## 4. Discussion

In this manuscript, we study the multifractal behavior of the geomagnetic field fluctuations at Swarm altitude for quiet (AE<200 nT) and active (AE>200 nT) conditions during one year (9 March 2016–9 March 2017) in both northern and southern hemisphere. One year provides a large enough set of spectra to be able to observe its spatial variation, as shown below. In future studies, we plan to analyze whether there are variations concerning other magnetospheric indexes (e.g., storms vs. substorms), solar wind parameters (P, V, B, dB, etc.), or even during the solar cycle. Data were obtained from the vector field magnetometer onboard Swarm A satellite, measured at low resolution (1 Hz). Using the *p*-leaders (with p=1) discrete wavelet MF, we obtained the spatial variation of three measures that quantitatively characterize the singularity spectrum of the magnetic fluctuations under quiet and active conditions, namely, the Hurst exponent *H*, $\xi_{2}$ that is related to the power-law index of the power spectra, and the relevance or strength of the multifractality of the magnetic fluctuations as characterized by the spectrum width ΔH. Our findings suggest that different turbulent regimes of the geomagnetic field fluctuations exist along the Swarm path.

The results for the northern hemisphere show that the value of *H* has a significant spatial variation, with some differences between quiet and active conditions. Larger values of *H* suggest a more persistent dynamic, while smaller values a more stochastic Brownian type of behavior. Hence, in general, at midnight the fluctuations are more persistent, while at dawn and dusk, they are more Brownian-like. Similarly, midday fluctuations become more persistent during active periods, compared to quiet times. Although the physical reason for these results deserves further study, it may provide clues to build future models that describe the ionospheric dynamics in these regions.

We see some interesting situations with negative values of *H*, suggesting the existence of behavior similar to wave breaking or shocklet-like propagating fronts. The physical processes corresponding to this behavior are interesting in themselves and could suggest some restrictions to detailed modeling efforts, not only about the magnetic field fluctuations, but also about the type of dynamics that can affect all the plasma variables.

The value of $\xi_{2}$, related to the power-law index of the magnetic field power spectrum, varies spatially, particularly between noon and midnight, with some changes between quiet and active times. Similarly, the relative importance of the strength of the multifractality in the intermittent behavior does not seem to change significantly between quiet and active times. However, their significance seems to be slightly higher for dawn and dusk, relative to noon and midnight.

The same analysis is repeated for the southern hemisphere. Although the multifractal analysis produced similar spatial patterns, the southern hemisphere seems to be more persistent (larger *H*) and slightly less multifractal (small ΔH) than the northern hemisphere, a result suggesting that the modeling of the northern hemisphere ionosphere may be more difficult than for the southern ionosphere.

Finally, we mention that many of the techniques that try to compute the multifractal spectrum, for example, those based on structure-functions that are normally used in turbulence, have trouble computing the D(h) for negative values of *q*. Hence, they have trouble obtaining the singularity spectrum for a reasonable range of *q* so that a proper extrapolation can be done, as it was done here. The calculation of ΔH is a new and relevant result since it provides a quantitative measure of the strength of the multifractality beyond the value of *H*. Remember that *H* characterizes the level of persistence (H>0.5), as opposed to regular Brownian motion (H<0). Therefore, the quantification of ΔH gives information about the distribution and strength of the expected fluctuations at a particular place and time, so that it provides useful insight to modeling efforts that can eventually evolve into usable space weather applications for magnetic field variations at ground-level, geophysical induced currents, etc. Such a working model must be able to accommodate the distribution of these fluctuations, which are different for monofractal and multifractal spectra so that the one with the longest tail will display a larger occurrence of stronger fluctuations. In turn, the relative importance of these large fluctuations may put some operating and design restrictions on electric networks that may be transmitting electricity in a particular region at a particular time, by the currents that are induced on them by these fluctuations. For example, operating under a 6-sigma reliability may not be sufficient for some of these distributions. Let us note that these fluctuations can be simulated with *p*-type of models of turbulence [59] so that we can test the operating restrictions of a particular design. Of course, to fully assess the effect of these geophysical induced currents on electric networks requires studying the time scales of the relevant regimes (scaling region in the MF), the local ground impedance, etc.

## Data Availability

Not Applicable.
